# Birth Weight and Diaphragmatic Ultrasound Measurements During the First 48 Hours in Term Neonates: A Secondary Longitudinal Analysis

**DOI:** 10.3390/diagnostics16193120

**Published:** 2026-09-25

**Authors:** Bogdan Rotea, Andra Rotea, Mădălina Ianca Suba, Ovidiu Rosca, Bogdan Cerbu, Ileana Enatescu, Mirabela Adina Dima, Emil Radu Iacob, Adrian Gluhovschi, Daniela Iacob

**Affiliations:** 1 Doctoral School, Victor Babeș University of Medicine and Pharmacy, 300041 Timișoara, Romania; andra.alecu@umft.ro (A.R.); madalina.suba@umft.ro (M.I.S.); bogdan.cerbu@umft.ro (B.C.); 2 Center for Multidisciplinary Research and Management in Pediatric Surgical Conditions, Iosif Nemoianu Street No. 2, 300011 Timișoara, Romania; enatescu.ileana@umft.ro (I.E.); dima.mirabela@umft.ro (M.A.D.); radueiacob@umft.ro (E.R.I.); iacob.daniela@umft.ro (D.I.); 3 Methodological and Infectious Diseases Research Center, Department of Infectious Diseases, Victor Babeș University of Medicine and Pharmacy, Eftimie Murgu Square No. 2, 300041 Timișoara, Romania; ovidiu.rosca@umft.ro; 4 Dr. Victor Babeș Infectious Diseases and Pneumononcology Hospital, 300310 Timișoara, Romania; 5 Department XIII, Discipline of Infectious Diseases, Victor Babeș University of Medicine and Pharmacy, Eftimie Murgu Square No. 2, 300041 Timișoara, Romania; 6 Discipline of Neonatology, Victor Babeș University of Medicine and Pharmacy, 300041 Timișoara, Romania; 7 Department of Pediatric Surgery and Orthopedics, Victor Babeș University of Medicine and Pharmacy, Eftimie Murgu Square No. 2, 300041 Timișoara, Romania; 8 Department XII, Discipline of Obstetrics and Gynecology, Victor Babeș University of Medicine and Pharmacy, 300041 Timișoara, Romania; adigluhovschi@yahoo.com

**Keywords:** birth weight, diaphragm thickness, diaphragmatic ultrasound, diaphragmatic thickening fraction, neonatal anthropometry, newborn

## Abstract

**Background/Objectives**: Neonatal diaphragm ultrasound measurements may vary with body size. We examined associations between birth weight and repeated bilateral diaphragm measurements during the first 48 h after birth. **Methods**: This exploratory secondary post hoc analysis included 116 term neonates assessed at approximately 1, 24, and 48 h (348 examinations). Associations of birth weight with diaphragm thickness, diaphragmatic thickening fraction (DTF), and excursion were estimated using generalized estimating equations adjusted for gestational age, sex, and assessment time. Mean bilateral end-expiratory thickness was a derived post hoc summary, and absolute inspiratory thickening (ΔT) was examined additionally. **Results**: Across the 12 exploratory birth-weight outcome tests, none remained below 0.05 after global Holm correction, and this global result is the primary summary of this analysis. The post hoc mean bilateral end-expiratory thickness summary showed a nominal positive association with birth weight (β = 0.0876 mm/kg; 95% CI, 0.0112–0.1640; nominal *p* = 0.0246; global Holm-adjusted *p* = 0.2465); this is reported as a descriptive, hypothesis-generating observation and not as the main finding. All four side-specific thickness coefficients were nominally positive, but none remained below 0.05 after global adjustment. Birth weight showed little evidence of association with excursion or ΔT. Right-sided DTF showed an inverse nominal association (β = −1.4779 percentage points/kg; 95% CI, −2.5933 to −0.3624; nominal *p* = 0.0094; global Holm-adjusted *p* = 0.1130); given unresolved DTF provenance uncertainty (see Methods), this estimate is not used to support any physiological conclusion. **Conclusions**: None of the 12 exploratory birth-weight associations remained below 0.05 after global Holm correction, and this negative global result, rather than any single nominal association, is the principal finding of this analysis. The post hoc bilateral thickness association is reported descriptively and should not be regarded as a main finding; ΔT and DTF are reported as auxiliary post hoc observations. These findings are incremental and hypothesis-generating and require prospective, prespecified validation.

## 1. Introduction

The transition from fetal to extrauterine life requires rapid establishment of pulmonary aeration and effective spontaneous ventilation, while respiratory mechanics continue to evolve during the first postnatal hours [1]. During this period, the diaphragm operates in a changing mechanical environment and can be assessed non-invasively at the bedside by ultrasonography. Diaphragm ultrasound provides several related but non-equivalent measurements: end-expiratory and end-inspiratory thickness describe muscle dimensions at different respiratory phases, excursion reflects diaphragmatic displacement, and diaphragmatic thickening fraction (DTF) expresses the relative inspiratory increase in thickness.

Neonatal diaphragm ultrasound has increasingly been studied in healthy newborns, with recent work providing reference values and reproducibility data for thickness, excursion, and DTF [2,3]. A recent neonatal point-of-care ultrasound review also highlighted ongoing challenges in protocol standardization, operator training, normative data, and validated clinical decision thresholds [4]. Longitudinal observations in healthy term neonates also suggest relative stability of several diaphragm measurements during the first postnatal days, although selected dynamic indices may vary with mode of delivery [5].

Body size is another potential determinant of absolute diaphragm dimensions. Rehan and McCool reported positive correlations between diaphragm thickness and birth weight, body length, and head circumference in healthy term infants [6]. In preterm infants, Alonso-Ojembarrena et al. reported that birth weight was the principal variable associated with diaphragm thickness at birth and during longitudinal follow-up [7]. More recently, Patti et al. reported positive associations of inspiratory and expiratory diaphragm thickness with birth weight in a neonatal cohort including infants with transient tachypnea and healthy controls, while excursion and DTF showed no clear relationship with weight [8]. De Macedo et al. further reported lower absolute inspiratory and expiratory thickness in small-for-gestational-age compared with appropriate-for-gestational-age term newborns, with attenuation of between-group differences after normalization by birth weight [9]. These studies establish that neonatal anthropometry can contribute to variation in absolute diaphragmatic thickness.

However, previous studies have been predominantly cross-sectional, and the association between continuous birth-weight variation and repeated bilateral diaphragm measurements during the immediate neonatal transition remains insufficiently characterized within an adjusted longitudinal framework. The source cohort used for the present analysis has previously contributed to reports on early lung ultrasound and subsequent oxygen requirement [10] and on serial diaphragm ultrasound trajectories during the first 48 h [11]. The former addressed lung ultrasound in relation to subsequent oxygen requirement, whereas the latter characterized temporal changes in diaphragm ultrasound measurements. Neither publication evaluated birth weight as a continuous exposure across repeated bilateral diaphragm measurements using adjusted longitudinal GEE models or reported the derived bilateral end-expiratory summary examined in the present exploratory analysis.

The objective of this exploratory secondary post hoc analysis was to quantify the association between birth weight and repeated bilateral diaphragmatic thickness measurements during the first 48 h after birth while accounting for gestational age, sex, postnatal assessment time, and within-neonate correlation. Associations with excursion and DTF were examined as secondary exploratory analyses. No prospectively prespecified anthropometric hypothesis was tested, and all analyses were considered hypothesis-generating.

## 2. Materials and Methods

### 2.1. Study Design and Participants

This was an exploratory secondary post hoc analysis of prospectively collected data from a single-center observational neonatal ultrasound cohort conducted in the Neonatology Department of the “Louis Turcanu” Children’s Hospital, Timisoara, Romania. The source cohort has been described previously [10,11]. The ultrasound examinations used in the present study overlap with those previous reports. The first publication evaluated early lung ultrasound in relation to subsequent oxygen requirement, whereas the second described serial temporal trajectories of diaphragm thickness, DTF, and excursion during the first 48 h. The present manuscript does not repeat those research questions. Instead, it reanalyzes the serial bilateral diaphragm measurements to estimate adjusted associations with birth weight as a continuous exposure using longitudinal GEE models, including birth-weight-by-time and robustness analyses that were not reported previously.

For the present analysis, records were included if gestational age was 37–42 completed weeks and linked bilateral diaphragm ultrasound assessments were available at approximately 1, 24, and 48 h. Of 121 source-cohort records, five with gestational age < 37 weeks were excluded. The analytical cohort comprised 116 term neonates and 348 repeated examinations.

### 2.2. Ultrasound Acquisition

Diaphragm ultrasound was performed at approximately 1 h after birth (target 60 ± 15 min), 24 h, and 48 h during quiet spontaneous breathing, with neonates supine or semi-recumbent, using a LOGIQ E10 system (GE HealthCare, Milwaukee, WI, USA).

Bilateral diaphragmatic thickness was measured with a 12 MHz linear transducer in the zone of apposition, approximately between the eighth and tenth intercostal spaces along the midaxillary region. End-expiratory and end-inspiratory values represented the mean of three technically acceptable respiratory cycles.

Bilateral diaphragmatic excursion was measured with a 3 MHz curvilinear transducer from a subcostal approach in M-mode, with the cursor aligned as perpendicular as technically feasible to diaphragmatic movement. Excursion was averaged across three technically acceptable cycles. All examinations were performed by a single sonographer. Formal intra- or inter-observer reproducibility testing was not performed.

### 2.3. Variables and Quality Control

Birth weight was the exposure of interest and was analyzed continuously in kilograms. Gestational age, sex, and categorical assessment time were covariates. Body length, head circumference, and chest circumference were examined separately as alternative exploratory anthropometric exposures.

Ultrasound outcomes were right and left end-expiratory thickness, end-inspiratory thickness, DTF, and excursion. Mean bilateral end-expiratory thickness was derived post hoc as the arithmetic mean of the right and left measurements at each assessment. It was selected as a single participant-level summary of bilateral resting diaphragm morphology, because end-expiratory thickness represents resting anatomical thickness and constitutes the denominator of DTF, without privileging either hemidiaphragm. It was not a prespecified primary outcome and was therefore treated explicitly as exploratory. An analogous bilateral end-inspiratory summary was not created because the secondary analysis was not intended to generate a parallel set of additional post hoc composite outcomes; side-specific end-inspiratory estimates were retained instead.

DTF was defined as DTF = (Tinsp − Texp)/Texp × 100. The originally recorded DTF values were used for the main analyses.

The reconciled database was checked for duplicate identifiers, duplicate participant–time combinations, assessment linkage, completeness of core variables, and plausibility of thickness and excursion measurements. Quality-control comparison of recorded DTF with values recalculated from stored mean thicknesses identified seven left-sided discrepancies, including three >4.5 percentage points. Averaging cycle-specific DTF is not mathematically identical to calculating DTF from averaged inspiratory and expiratory thickness values and could potentially explain such discrepancies; however, this could not be verified because cycle-level source measurements were unavailable. Recorded DTF was therefore retained to preserve the original dataset, whereas recalculated DTF was used only as a QC/sensitivity analysis. Because the origin of these discrepancies could not be confirmed and cycle-level measurements are no longer available, DTF provenance remains uncertain; DTF results in this analysis are accordingly reported strictly as descriptive, exploratory observations and are not used to support any physiological conclusion.

Following examination of the DTF findings, absolute inspiratory thickening was additionally defined as ΔT = Tinsp − Texp. Because ΔT was conceived only after the DTF results had been seen, right-sided, left-sided, and bilateral mean ΔT are presented strictly as an auxiliary post hoc analysis and are not used to support conclusions independent of that context.

### 2.4. Statistical Analysis

Continuous variables were summarized as mean ± standard deviation or median [interquartile range], as appropriate, and categorical variables as counts and percentages.

Longitudinal associations were estimated using Gaussian generalized estimating equations (GEE) with an identity link, participant-level clustering, an exchangeable working-correlation structure, and robust sandwich covariance estimates [12]. The base exploratory model included birth weight, gestational age, sex, and categorical assessment time. Coefficients represent adjusted population-averaged differences in the outcome per 1 kg higher birth weight and are reported with 95% confidence intervals. Working-correlation robustness was assessed by refitting the base exploratory model using independence and first-order autoregressive [AR(1)] structures.

The same model specification was applied to the eight side-specific ultrasound outcomes and to the three additional ΔT outcomes. Because all analyses were post hoc and several derived outcomes were examined, the global multiplicity approach treated all 12 birth-weight outcome tests as a single exploratory family: mean bilateral end-expiratory thickness; right and left end-expiratory thickness; right and left end-inspiratory thickness; right and left DTF; right and left excursion; and right-sided, left-sided, and bilateral ΔT. Nominal birth-weight *p* values were adjusted using the Holm procedure [13]. This global Holm correction is a retrospective sensitivity analysis rather than a substitute for prespecification: the outcome family itself was assembled after the data had been examined, and it includes outcomes—the bilateral end-expiratory summary and the three ΔT outcomes—that were constructed only after preliminary results had been seen. Applying a multiplicity correction after such outcomes have been defined can quantify the number of tests performed but cannot fully remove the analytic flexibility introduced by data-driven selection and construction of outcomes. Effect estimates and 95% confidence intervals were emphasized throughout, and multiplicity-adjusted *p* values were used only to contextualize the exploratory evidence rather than to define confirmatory findings.

The ΔT analyses used the same GEE specification. Because ΔT was introduced after examination of the DTF findings, right-sided, left-sided, and bilateral ΔT were explicitly regarded as additional post hoc outcomes and were included in the global 12-outcome Holm sensitivity framework described above.

Mode of delivery was not included in the base exploratory adjustment set because it was not assumed to be a pure baseline confounder: delivery mode may itself reflect fetal size and obstetric indications related to birth weight. Given evidence that delivery mode can influence early neonatal diaphragm measurements [5], it was evaluated in a dedicated sensitivity model; only one neonate lacked delivery-mode data. Additional sensitivity analyses included a quadratic birth-weight term, birth-weight-by-time interaction, alternative independence and first-order autoregressive working-correlation structures, and leave-one-neonate-out analysis. Pearson residuals were reviewed descriptively. Length, head circumference, and chest circumference were evaluated separately in comparable exploratory models and were not used to support significance-based conclusions regarding birth weight.

No imputation was performed because variables in the base exploratory model were complete. No retrospective power calculation was undertaken. Analyses were performed in Python 3.13.5 (Python Software Foundation, Beaverton, OR, USA) using statsmodels 0.14.6. Reporting was guided by the STROBE recommendations for observational studies [14].

### 2.5. Ethics, Data Protection

The parent study was conducted in accordance with the Declaration of Helsinki and was approved by the Institutional Ethics Committee of “Pius Brinzeu” Emergency County Hospital Timisoara (approval no. 585, 16 December 2025). Written informed consent was obtained from parents or legal guardians before enrollment in the parent study. The present secondary analysis required no additional participant contact or ultrasound examination.

The working dataset was coded and maintained under restricted access. No directly identifying information was included in analytical outputs. The post hoc nature of the anthropometric analyses and overlap of the source cohort with previous publications are explicitly disclosed.

## 3. Results

### 3.1. Cohort Characteristics

Of 121 source-cohort records screened, five neonates with gestational age < 37 completed weeks were excluded. The analytical cohort therefore comprised 116 term neonates, each with bilateral diaphragm ultrasound assessments at approximately 1, 24, and 48 h, yielding 348 longitudinal observations.

Mean gestational age was 38.72 ± 1.12 weeks and mean birth weight was 3300.5 ± 472.6 g; 62 neonates (53.4%) were male. Mean length was 51.37 ± 2.51 cm, head circumference 34.58 ± 1.67 cm, and chest circumference 33.42 ± 2.21 cm. Delivery mode was available for 115 neonates, of whom 92 (80.0%) were delivered by cesarean section (Table 1). Birth weight, gestational age, sex, and core diaphragm outcomes were complete for the base exploratory analysis.

Supplementary oxygen use was captured only as a binary indicator at each scheduled assessment. Oxygen was recorded as being administered in 26 of 116 neonates (22.4%) at the 1 h assessment, in 1 of 116 (0.9%) at 24 h, and in 0 of 116 (0%) at 48 h. The database did not allow determination of the exact timing of oxygen initiation or discontinuation relative to each ultrasound assessment, and it did not contain usable data on continuous positive airway pressure, non-invasive or invasive ventilation, formal respiratory diagnosis, or neonatal intensive care unit admission. These oxygen data are reported descriptively only; they were not modeled as a covariate or confounder in the present analysis.

### 3.2. Birth Weight and Diaphragm Thickness

In the adjusted GEE model, higher birth weight showed a positive association with mean bilateral end-expiratory diaphragm thickness, with an estimated population-averaged difference of 0.0876 mm per 1 kg higher birth weight (95% CI, 0.0112–0.1640; nominal *p* = 0.0246) (Table 2; Supplementary Figure S3). Because this bilateral summary was derived post hoc, its nominal *p* value was interpreted as exploratory. Under the global Holm correction across all 12 exploratory birth-weight outcome tests, its adjusted *p* value was 0.2465, and no outcome remained below the 0.05 threshold.

All four side-specific thickness coefficients were positive: 0.0999 mm/kg for right end-expiratory thickness (95% CI, 0.0142–0.1856; nominal *p* = 0.0224), 0.0753 mm/kg for left end-expiratory thickness (95% CI, −0.0031 to 0.1536; nominal *p* = 0.0597), 0.0883 mm/kg for right end-inspiratory thickness (95% CI, −0.0094 to 0.1859; nominal *p* = 0.0766), and 0.0727 mm/kg for left end-inspiratory thickness (95% CI, −0.0184 to 0.1638; nominal *p* = 0.1178) (Figure 1). None remained below the 0.05 threshold after the global Holm correction.

### 3.3. Dynamic Ultrasound Indices

In the additional post hoc analysis, birth weight showed little evidence of association with absolute inspiratory thickening. The adjusted coefficient was −0.0116 mm/kg on the right (95% CI, −0.0373 to 0.0140; nominal *p* = 0.3737), −0.0026 mm/kg on the left (95% CI, −0.0237 to 0.0186; nominal *p* = 0.8128), and −0.0071 mm/kg for bilateral mean ΔT (95% CI, −0.0284 to 0.0142; nominal *p* = 0.5130) (Table 3). All three global Holm-adjusted *p* values were 1.0000.

Right-sided DTF showed an inverse nominal association with birth weight (β = −1.4779 percentage points/kg; 95% CI, −2.5933 to −0.3624; nominal *p* = 0.0094), but the global Holm-adjusted *p* value was 0.1130. Left-sided DTF showed a smaller and statistically uncertain inverse estimate (β = −0.7769 percentage points/kg; 95% CI, −1.6052 to 0.0514; nominal *p* = 0.0660; global Holm-adjusted *p* = 0.5375). Birth weight was not clearly associated with diaphragmatic excursion, and both excursion estimates had global Holm-adjusted *p* values of 1.0000. Sensitivity analysis using DTF recalculated from the stored mean thickness values did not materially alter the overall interpretation.

### 3.4. Sensitivity and Robustness Analyses

Given the marked predominance of cesarean delivery, mode of delivery was examined explicitly in a sensitivity model. Additional adjustment for delivery mode produced an almost unchanged estimate for mean bilateral end-expiratory thickness (β = 0.0845 mm/kg; 95% CI, 0.0106–0.1583; nominal *p* = 0.0250; *n* = 115) compared with the base exploratory model (β = 0.0876 mm/kg). There was little evidence of marked nonlinearity (quadratic term *p* = 0.8892) or birth-weight-by-time interaction (joint *p* = 0.8745) (Table 4).

The base exploratory estimate was unchanged under an independence working correlation (β = 0.0876 mm/kg; 95% CI, 0.0112–0.1640) and similar using AR(1) correlation (β = 0.0898 mm/kg; 95% CI, 0.0121–0.1675). Residual review showed no evident fitted-value pattern (Supplementary Table S3 and Supplementary Figure S1).

Leave-one-neonate-out coefficients remained positive in every iteration (0.0759–0.1097 mm/kg); however, statistical inference was not stable: nominal *p* values ranged from 0.0007 to 0.0660, and in at least one iteration the 95% confidence interval crossed zero. Thus, while the direction of the point estimate was stable, the statistical significance of the association was not robust to exclusion of individual neonates.

Alternative anthropometric analyses showed no clear association for length or chest circumference. Head circumference showed a nominal positive association with bilateral end-expiratory thickness (β = 0.0233 mm/cm; 95% CI, 0.0028–0.0439; *p* = 0.0261), but this exploratory result was not interpreted as confirmatory.

Sensitivity and alternative anthropometric analyses were exploratory. Their nominal *p* values are descriptive and are not part of the global 12-outcome birth-weight Holm family.

## 4. Discussion

### 4.1. Principal Findings

In this exploratory secondary post hoc longitudinal analysis of term neonates, no birth-weight association with diaphragm ultrasound measurements remained below the 0.05 threshold after global Holm correction across all 12 exploratory outcome tests, and this global result is the principal finding. Birth weight showed a consistent positive direction of association with absolute diaphragm thickness across both hemidiaphragms and respiratory phases, and the derived post hoc bilateral end-expiratory measure increased by approximately 0.088 mm per 1 kg higher birth weight after adjustment for gestational age, sex, and assessment time; because this bilateral outcome was constructed after the data had been examined, its nominal association is reported descriptively and should not be interpreted as confirmatory, and global multiplicity correction—itself only a retrospective sensitivity check—does not resolve that limitation. Birth weight showed little evidence of association with diaphragmatic excursion or absolute inspiratory thickening.

### 4.2. Anthropometry and Diaphragmatic Morphology

The direction of the present findings is consistent with previous neonatal observations. Rehan and McCool found positive relationships between diaphragm thickness and birth weight, body length, and head circumference in healthy term infants [6]. Patti et al. subsequently reported positive relationships between birth weight and inspiratory and expiratory diaphragm thickness [8], while de Macedo et al. observed lower absolute thickness among small-for-gestational-age compared with appropriate-for-gestational-age term newborns [9].

The present study extends this literature by evaluating birth weight continuously across repeated bilateral assessments during the first 48 h while accounting for gestational age, sex, assessment time, and within-neonate correlation. The estimated coefficient corresponds to approximately 0.0438 mm greater bilateral end-expiratory thickness per 500 g higher birth weight. This difference is quantitatively modest. Published neonatal studies have reported generally favorable intra-rater reliability for diaphragm thickness, with inter-rater performance varying according to protocol and measurement conditions [2,3]. However, reliability coefficients from other studies cannot define an absolute repeatability threshold for the operator-device combination used here. Because no intra- or inter-observer reproducibility study was performed in this cohort, we cannot determine whether a difference of approximately 0.044 mm per 500 g exceeds protocol-specific measurement variability. No validated neonatal threshold currently establishes such a difference as clinically meaningful, and without a reproducibility study for this operator-device combination, the biological relevance of this effect size cannot be established; the observed difference may lie within the range of ordinary measurement variability for this protocol, and no claim of biological or clinical significance is made for it.

Exploratory results for alternative anthropometric measures were heterogeneous. Head circumference showed a nominal positive association, whereas length and chest circumference did not. This does not establish head circumference as a superior anthropometric determinant, particularly because these analyses were exploratory and the original measurement procedures for these variables were not documented in sufficient detail to quantify measurement error.

The present findings do not justify simple normalization of diaphragm thickness by body weight. The study did not prespecify or validate a proportional scaling relationship between diaphragm thickness and body weight; therefore, expressing thickness as a simple ratio per kilogram could introduce assumptions that were not tested in this cohort. Prospective studies specifically designed to characterize body-size scaling are required before weight-normalized reference values can be recommended.

### 4.3. Dynamic Indices and DTF

Absolute inspiratory thickening provides useful context for the DTF findings. Although expiratory and inspiratory thickness coefficients were consistently positive, birth weight showed little evidence of association with the absolute inspiratory change in thickness.

DTF is defined as the absolute inspiratory change divided by expiratory thickness. A larger expiratory thickness without a proportional increase in absolute thickening can therefore mathematically produce a lower relative DTF. This provides a possible explanation for the observed pattern, but the present analysis did not directly test denominator scaling as a physiological mechanism.

The inverse right-sided DTF association was nominal and did not remain below the 0.05 threshold after global Holm correction. It should therefore not be interpreted as evidence of reduced diaphragmatic performance in heavier neonates.

Interpretation is further limited by measurement and calculation-provenance considerations. Reliability of DTF varies across neonatal ultrasound protocols [2,3], and several recorded left-sided DTF values differed from values recalculated from stored mean thicknesses. Because cycle-level source measurements were unavailable, the origin of these discrepancies cannot be confirmed. This unresolved provenance uncertainty means DTF findings in this analysis, including the right-sided inverse association described above, are not used to support any physiological conclusion and are reported descriptively only.

Birth weight also showed little evidence of association with excursion. Neither excursion nor DTF should be regarded as a direct measurement of muscle strength; both reflect interactions among respiratory effort, respiratory-system mechanics, and measurement conditions.

### 4.4. Early Postnatal Transition

The first 48 h after birth represent a period of continuing adaptation in lung aeration and respiratory mechanics [1]. In the previously published longitudinal analysis of this cohort, changes in diaphragm ultrasound parameters were comparatively small during this interval [11]. The present study should be regarded as an incremental secondary re-analysis of the same serial ultrasound measurements rather than the identification of a new diaphragmatic biomarker; its novelty lies in the anthropometric question and in the longitudinal modeling of birth weight as a continuous exposure, not in new measurements or a newly discovered physiological marker.

The present analysis addresses a different question: whether between-neonate differences in birth weight are associated with repeated diaphragm measurements during this transition. No clear birth-weight-by-time interaction was detected for the bilateral end-expiratory outcome. This is compatible with a relatively stable estimated association across the three measurements but does not establish temporal invariance, particularly because the study was not designed specifically to detect small interaction effects.

Mode of delivery was not treated as a pure baseline confounder in the base model because it may itself reflect fetal size and obstetric indications related to birth weight. Nevertheless, it is clinically relevant because delivery mode may influence early neonatal diaphragm parameters [5]. A dedicated sensitivity model therefore added delivery mode to the base adjustment set; the bilateral end-expiratory coefficient changed only slightly (0.0845 versus 0.0876 mm/kg). This supports stability of the estimate to delivery-mode adjustment, while the marked predominance of cesarean delivery still limits precise assessment of delivery-related differences or effect modification.

### 4.5. Strengths and Limitations

Strengths include prospectively collected data, a standardized bilateral ultrasound protocol, three scheduled assessments per neonate, complete base-model data, and longitudinal analysis accounting for within-neonate correlation. The analytical hierarchy was explicitly exploratory, and the bilateral end-expiratory coefficient remained similar across alternative working-correlation assumptions and after delivery-mode adjustment.

Several limitations remain. The study was single-center, secondary, and post hoc, and no sample-size calculation was performed for the anthropometric question. Generalizability is limited to clinically stable term neonates, and these findings should not be extrapolated directly to preterm infants. Recent studies in preterm neonates have demonstrated gestational-age-, birth-weight-, respiratory-support-, and diaphragm-function-related differences, supporting separate evaluation of preterm populations [7,15,16,17]. Formal intra- and inter-observer reproducibility testing was not undertaken, which is particularly relevant given the small magnitude of the estimated thickness difference. Feeding status, behavioral state, and direct measures of respiratory effort were not recorded. Clinical respiratory characterization of the cohort was also limited: beyond a binary oxygen indicator at each assessment (present in 26/116 neonates at 1 h, 1/116 at 24 h, and 0/116 at 48 h), the database did not permit determination of the exact timing of oxygen use and contained no usable data on continuous positive airway pressure, non-invasive or invasive ventilation, formal respiratory diagnosis, or neonatal intensive care unit admission; respiratory status could therefore not be formally evaluated as a determinant or confounder of diaphragm measurements. Gestational age was available in completed weeks, and the dataset was not designed to identify fetal growth restriction. DTF was additionally subject to the calculation-provenance issues described above; because the origin of the recorded-versus-recalculated discrepancies cannot be confirmed, DTF results are not used to support physiological conclusions. Mean bilateral end-expiratory thickness was a derived post hoc summary rather than a prespecified outcome, and its use as the paper’s central result carries an inherent risk of outcome-selection bias that explicit post hoc labeling and multiplicity correction cannot fully remove. Its nominal association should therefore not be interpreted as confirmatory; after global Holm correction across all 12 exploratory birth-weight outcome tests, its adjusted *p* value was 0.2465, and no association in this family remained significant. The leave-one-neonate-out analysis showed a positive coefficient in every iteration (0.0759 to 0.1097 mm/kg), but statistical inference was not stable: nominal *p* values ranged from 0.0007 to 0.0660, and in at least one iteration, the 95% confidence interval crossed zero (lower bound as low as −0.0050). Thus, although the direction of the point estimate was consistently positive, its statistical significance depended materially on individual observations and should not be regarded as robust, particularly in a cohort of only 116 neonates. Finally, residual confounding remains possible, and the observational design precludes causal inference.

### 4.6. Implications for Ultrasound Interpretation

The present findings are primarily relevant to interpretation and development of future reference models rather than immediate bedside decision-making. Neonatal body size may contribute to variability in absolute diaphragm thickness, but these exploratory data do not support weight-adjusted diagnostic thresholds or simple expression of diaphragm thickness per kilogram.

Prospective multicenter studies should prespecify anthropometric exposures and ultrasound outcomes, incorporate a wider range of neonatal body sizes and growth phenotypes, standardize behavioral and feeding state, preserve cycle-level measurements, and formally assess measurement reproducibility. External validation would be required before anthropometry could be incorporated into neonatal diaphragm reference equations or clinical decision thresholds.

## 5. Conclusions

None of the 12 exploratory birth-weight outcome tests remained below the 0.05 threshold after global Holm correction, and this negative global result is the principal finding of this analysis. Higher birth weight showed a consistent positive direction of association with absolute diaphragm thickness across repeated assessments, but the bilateral summary outcome that carried this association was derived post hoc, is reported here descriptively, and should not be read as a confirmed main finding; global Holm correction is a retrospective sensitivity check and does not by itself resolve the analytic flexibility introduced by selecting and constructing outcomes after the data had been examined. Associations with diaphragmatic excursion were less evident, and ΔT and DTF are reported only as auxiliary, post hoc observations; unresolved DTF provenance uncertainty means that the inverse right-sided DTF association does not support any physiological conclusion. This work is an incremental secondary re-analysis of an already studied cohort, and its novelty lies in the anthropometric question and the longitudinal modeling of birth weight as a continuous exposure, not in a new diaphragmatic biomarker. Overall, these findings are hypothesis-generating and require prospective, prespecified validation. They do not support simple weight normalization or weight-adjusted diagnostic thresholds for neonatal diaphragm ultrasound.

## Figures and Tables

**Figure 1:**
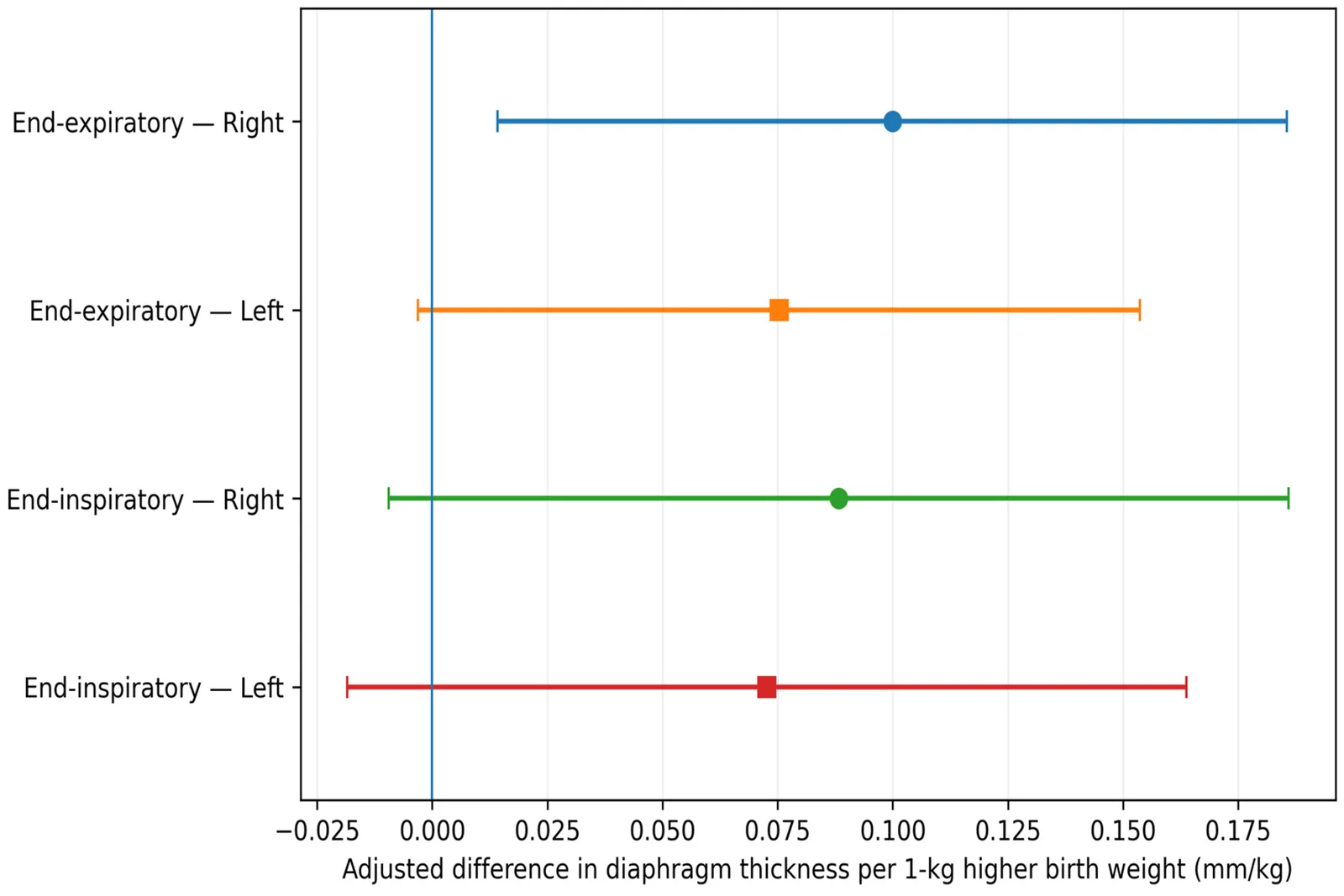
Side-specific associations between birth weight and end-expiratory and end-inspiratory diaphragm thickness. Markers indicate adjusted coefficients per 1 kg higher birth weight and horizontal bars indicate 95% confidence intervals. Right and left hemidiaphragm estimates are labeled directly. Global Holm-adjusted *p* values across the 12 exploratory birth-weight outcome tests are reported in Table 2.

**Table 1:** Baseline characteristics of the analytical cohort.

Characteristic	Available *n*	Value
Gestational age, weeks	116	38.72 ± 1.12
Birth weight, g	116	3300.5 ± 472.6
Male sex	116	62 (53.4%)
Length, cm	116	51.37 ± 2.51
Head circumference, cm	116	34.58 ± 1.67
Chest circumference, cm	116	33.42 ± 2.21
Apgar score at 1 min	116	9 [8–9]
Apgar score at 5 min	95	10 [10–10]
Cesarean delivery	115	92 (80.0%)

Values are mean ± SD, median [interquartile range], or *n* (%), as appropriate.

**Table 2:** Adjusted association between birth weight and diaphragmatic thickness.

Outcome	*n*	Observations	β per 1 kg Higher Birth Weight (mm/kg)	95% CI	Nominal *p*	Global Holm *p*
Mean bilateral end-expiratory thickness *	116	348	0.0876	0.0112 to 0.1640	0.0246	0.2465
Right end-expiratory thickness	116	348	0.0999	0.0142 to 0.1856	0.0224	0.2462
Left end-expiratory thickness	116	348	0.0753	−0.0031 to 0.1536	0.0597	0.5375
Right end-inspiratory thickness	116	348	0.0883	−0.0094 to 0.1859	0.0766	0.5375
Left end-inspiratory thickness	116	348	0.0727	−0.0184 to 0.1638	0.1178	0.7067

Gaussian generalized estimating equations adjusted for gestational age, sex, and categorical assessment time. Coefficients are expressed in mm/kg. Global Holm *p* values refer to a single multiplicity sensitivity family comprising all 12 exploratory birth-weight outcome tests (the bilateral end-expiratory summary, eight side-specific ultrasound outcomes, and three ΔT outcomes). * Mean bilateral end-expiratory thickness was derived post hoc as the arithmetic mean of right and left end-expiratory thickness at each assessment.

**Table 3:** Adjusted associations of birth weight with dynamic diaphragmatic indices.

Outcome	*n*	Observations	β per 1 kg Higher Birth Weight	95% CI	Nominal *p*	Global Holm *p*
Right absolute inspiratory thickening, ΔT	116	348	−0.0116 mm	−0.0373 to 0.0140	0.3737	1.0000
Left absolute inspiratory thickening, ΔT	116	348	−0.0026 mm	−0.0237 to 0.0186	0.8128	1.0000
Mean bilateral ΔT *	116	348	−0.0071 mm	−0.0284 to 0.0142	0.5130	1.0000
Right DTF, original value	116	348	−1.4779 pp	−2.5933 to −0.3624	0.0094	0.1130
Left DTF, original value	116	348	−0.7769 pp	−1.6052 to 0.0514	0.0660	0.5375
Right excursion	116	348	+0.0808 mm	−0.0827 to 0.2442	0.3328	1.0000
Left excursion	116	348	+0.0824 mm	−0.0724 to 0.2373	0.2969	1.0000

For ΔT and excursion, coefficients are expressed in mm/kg; for DTF, coefficients are expressed in percentage points/kg. CI = confidence interval; DTF = diaphragmatic thickening fraction; ΔT = absolute inspiratory thickening. Global Holm *p* values refer to the 12-outcome exploratory birth-weight family defined in Methods. * Derived bilateral ΔT was introduced after examination of the DTF findings.

**Table 4:** Sensitivity, robustness, and alternative anthropometric analyses.

Analysis	*n*	Estimate/Statistic	95% CI	Nominal *p*	Interpretive Role
Base exploratory model: birth weight + gestational age + sex + assessment time	116	0.0876 mm/kg	0.0112 to 0.1640	0.0246	Base exploratory estimate
Additional delivery-mode adjustment	115	0.0845 mm/kg	0.0106 to 0.1583	0.0250	Delivery-mode sensitivity
Independence working correlation	116	0.0876 mm/kg	0.0112 to 0.1640	0.0246	Sensitivity
AR(1) working correlation	116	0.0898 mm/kg	0.0121 to 0.1675	0.0235	Sensitivity
Quadratic birth-weight term	116	Quadratic term	—	0.8892	No clear evidence of marked nonlinearity
Birth weight × time interaction	116	Joint Wald test	—	0.8745	No clear evidence of time modification
Leave-one-neonate-out	115	β 0.0759–0.1097 mm/kg	Lower CI bound reached −0.0050	0.0007–0.0660	Direction stable; precision moderately sensitive
Length, per cm	116	0.0085 mm/cm	−0.0054 to 0.0224	0.2291	Exploratory
Head circumference, per cm	116	0.0233 mm/cm	0.0028 to 0.0439	0.0261	Exploratory; nominal *p* only
Chest circumference, per cm	116	0.0015 mm/cm	−0.0174 to 0.0204	0.8770	Exploratory

## Data Availability

The data presented in this study are available on reasonable request from the corresponding author. The data are not publicly available because they concern neonatal participants and are subject to ethical, institutional, and data-protection restrictions.

## Supplementary Materials

The following supporting information can be downloaded at: https://www.mdpi.com/article/10.3390/diagnostics16193120/s1, Table S1: Serial diaphragm ultrasound measurements at 1, 24, and 48 h; Table S2: DTF calculation-provenance audit; Table S3: Base exploratory GEE model diagnostics and robustness checks; Figure S1: Pearson residuals versus fitted values; Figure S2: Normal Q-Q plot of Pearson residuals; Figure S3: Adjusted association between birth weight and mean bilateral end-expiratory diaphragm thickness.
