# Supplementary material for: Birth Weight and Diaphragmatic Ultrasound Measurements During the First 48 Hours in Term Neonates: A Secondary Longitudinal Analysis
Source: Diagnostics (Basel). 2026 Sep 25;16(19):3120. doi: 10.3390/diagnostics16193120 (PMC13664031; doi:10.3390/diagnostics16193120)
Supplement: Supplementary file 1 [file diagnostics-16-03120-s001.zip › diagnostics-4589923-supplementary.pdf]

Supplementary Material

Birth Weight and Diaphragmatic Ultrasound Measurements During the First 48 Hours in Term Neonates: A Secondary Longitudinal Analysis

Bogdan Rotea et al.

Supplementary material for submission to Diagnostics (MDPI).

Supplementary Table S1. Serial diaphragm ultrasound measurements at 1, 24, and 48 h

Outcome	1 h	24 h	48 h
Right end-expiratory thickness, mm	2.243 ± 0.271	2.180 ± 0.251	2.259 ± 0.300
Left end-expiratory thickness, mm	2.238 ± 0.281	2.188 ± 0.267	2.250 ± 0.284
Right end-inspiratory thickness, mm	2.708 ± 0.282	2.662 ± 0.281	2.726 ± 0.336
Left end-inspiratory thickness, mm	2.716 ± 0.302	2.674 ± 0.304	2.736 ± 0.326
Right DTF, %	20.988 ± 3.899	22.333 ± 5.101	20.910 ± 3.944
Left DTF, %	21.603 ± 3.961	22.434 ± 4.696	21.760 ± 3.780
Right excursion, mm	5.652 ± 0.698	5.785 ± 0.622	5.896 ± 0.593
Left excursion, mm	5.648 ± 0.675	5.797 ± 0.618	5.909 ± 0.545
Right ΔT, mm	0.465 ± 0.070	0.482 ± 0.100	0.467 ± 0.084
Left ΔT, mm	0.478 ± 0.073	0.486 ± 0.096	0.486 ± 0.086
Mean bilateral ΔT, mm	0.472 ± 0.057	0.484 ± 0.084	0.477 ± 0.075

Values are mean ± SD in the analytical cohort (n=116 at each time point). DTF values are the original recorded values. These summaries are descriptive; formal temporal trajectory testing was reported in the parent longitudinal publication.

Supplementary Table S2. DTF calculation-provenance audit

Record	Time	Side	Original DTF (%)	Recalculated DTF (%)	Difference (pp)
QC-01	48h	Left	22.7879	22.7273	-0.0606
QC-02	1h	Left	19.2308	23.8095	+4.5788
QC-03	24h	Left	14.2857	19.0476	+4.7619
QC-04	48h	Left	22.2727	22.7273	+0.4545
QC-05	24h	Left	23.8095	19.2308	-4.5788
QC-06	24h	Left	22.2727	22.7273	+0.4545
QC-07	48h	Left	22.2727	22.7273	+0.4545

These differences do not establish transcription error. Cycle-specific DTF averaging and DTF calculated from averaged inspiratory and expiratory thicknesses are not mathematically identical. Original participant identifiers are not shown.

Supplementary Table S3. Base exploratory GEE model diagnostics and robustness checks

Panel A. Working-correlation sensitivity

Structure	β BW (mm/kg)	95% CI	p	Correlation parameter
Independence	0.0876	0.0112 to 0.1640	0.0246	—
Exchangeable	0.0876	0.0112 to 0.1640	0.0246	0.3391
AR(1)	0.0898	0.0121 to 0.1675	0.0235	0.4092

Panel B. Residual diagnostics

Diagnostic	Value	Interpretation
Pearson residual mean	0.0000	Centered residuals
Pearson residual SD	0.9928	Residual dispersion
Pearson residual skewness	0.6959	Descriptive only
Pearson residual excess kurtosis	0.5821	Descriptive only

|Pearson residual| >2	15	Potentially unusual observations
|Pearson residual| >3	2	More extreme observations
Correlation: residual vs fitted	0.0000	No strong mean-pattern signal if near 0
Correlation: |residual| vs fitted	-0.0001	No strong variance-pattern signal if near 0

Panel C. Leave-one-neonate-out influence analysis

Metric	Value	Interpretation
Reference β	0.0876 mm/kg	Exchangeable GEE focal estimate
Minimum β after omission	0.0759 mm/kg	All coefficients remained positive
Maximum β after omission	0.1097 mm/kg	All coefficients remained positive
Maximum absolute change in β	0.0221 mm/kg	Largest single-neonate influence
Nominal p-value range	0.0007–0.0660	Direction stable; threshold crossing not invariant

Across independence, exchangeable, and AR(1) working-correlation assumptions, the focal coefficient was materially unchanged. Residual summaries are descriptive and are not treated as formal normality requirements for robust GEE inference.

Supplementary Figure S1. Pearson residuals versus fitted values

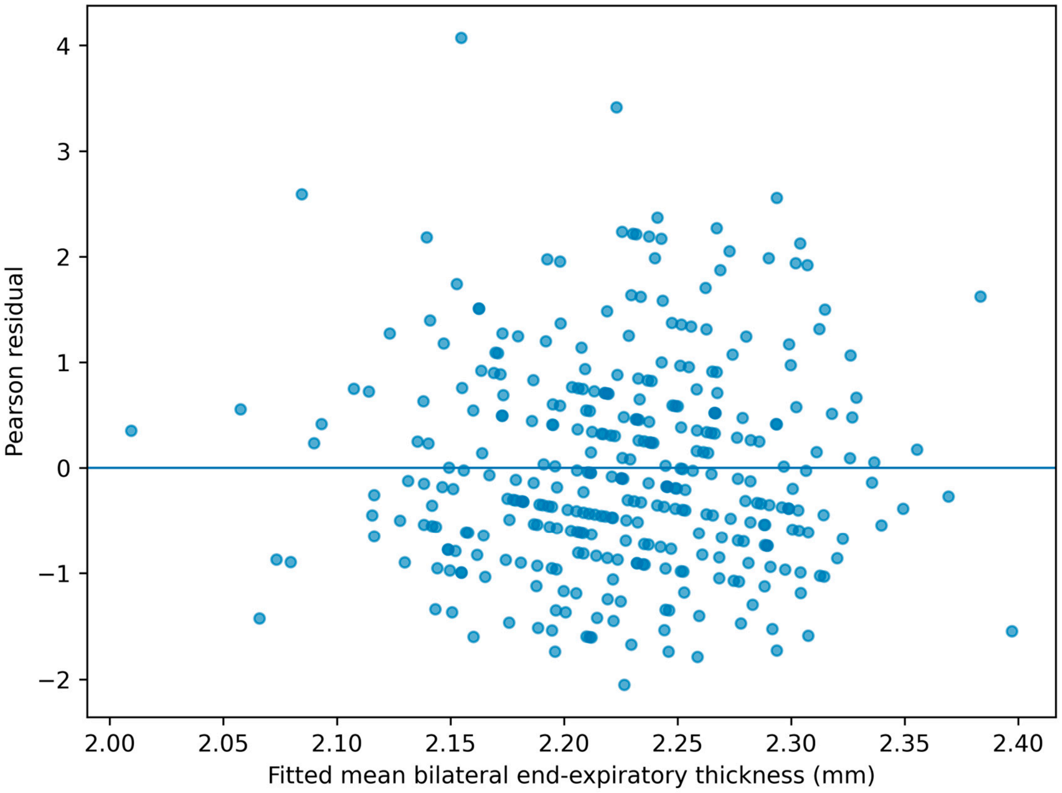

Pearson residuals from the focal exchangeable GEE model plotted against fitted mean bilateral end-expiratory thickness. No strong relationship between residual magnitude and fitted values was evident.

Supplementary Figure S2. Normal Q-Q plot of Pearson residuals

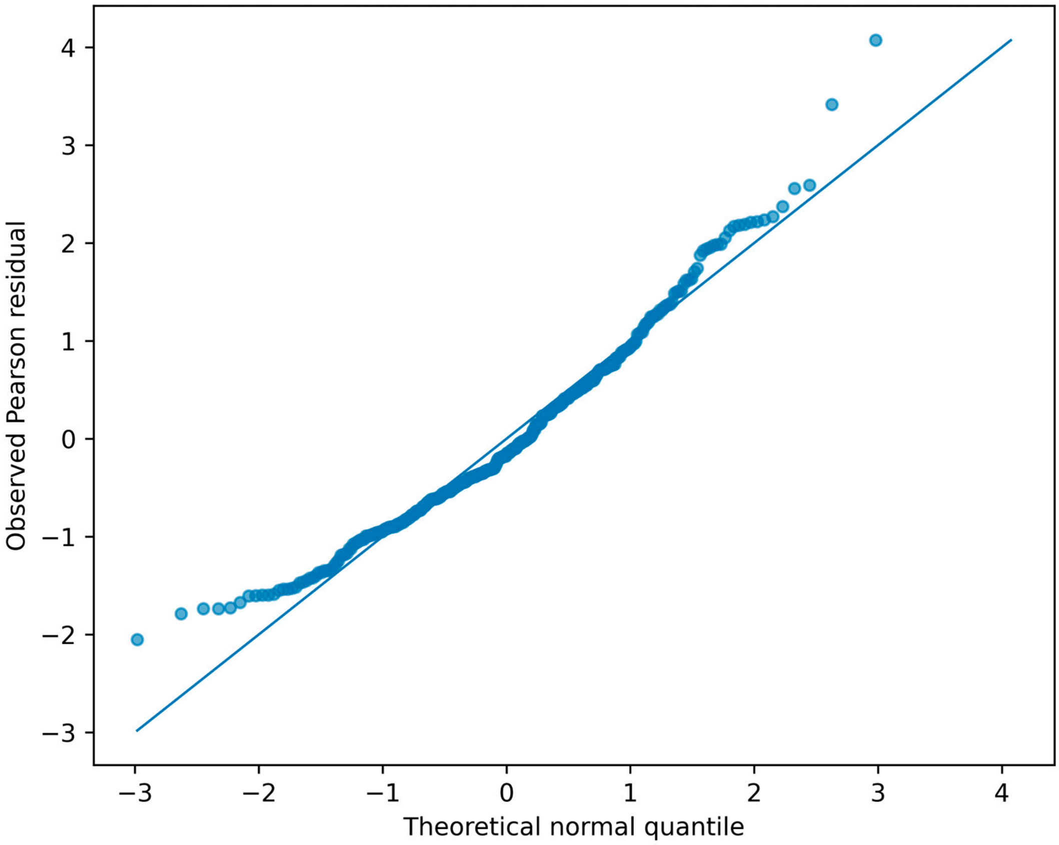

Normal Q-Q plot provided to characterize residual shape. Departures in the upper tail are visible; normality was not used as a validity criterion for robust GEE inference.

Supplementary Figure S3. Adjusted association between birth weight and mean bilateral end-expiratory diaphragm thickness

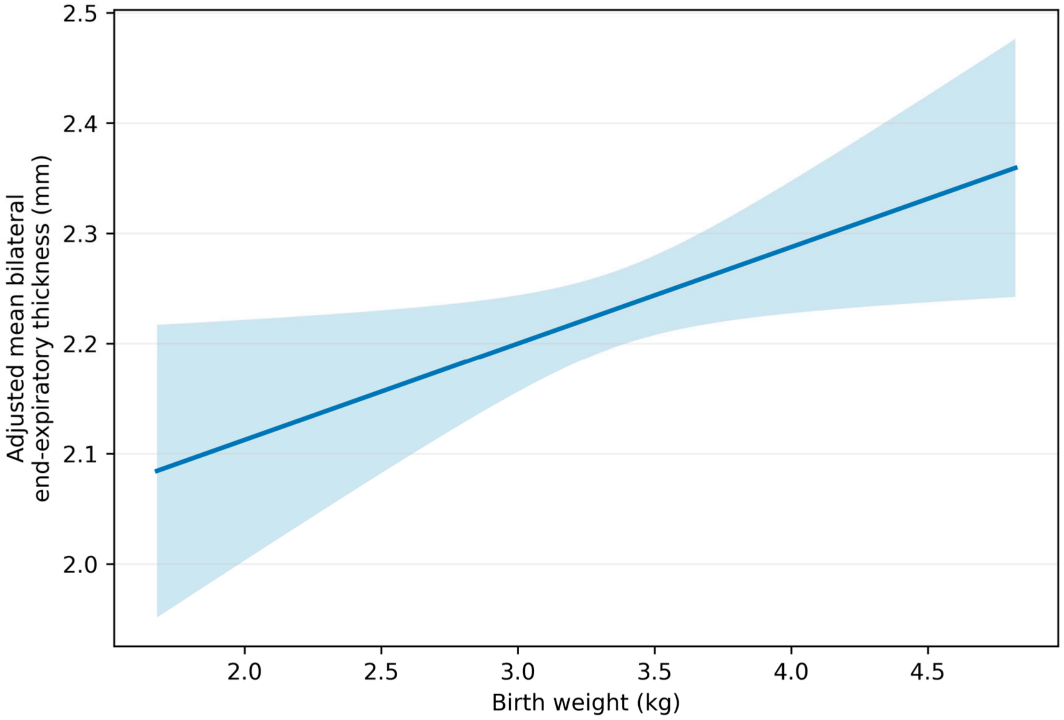

The solid line shows the model-based population-averaged prediction from the base exploratory model, averaged across the three assessment times and standardized to the empirical gestational-age and sex distribution. The shaded band denotes the 95% confidence interval. Mean bilateral end-expiratory thickness was a derived post hoc exploratory summary outcome; this figure is provided in the Supplementary Materials because the underlying outcome was not prespecified and did not remain significant after global multiplicity correction.
